# *Mammaliicoccus* spp. from German Dairy Farms Exhibit a Wide Range of Antimicrobial Resistance Genes and Non-Wildtype Phenotypes to Several Antibiotic Classes

**DOI:** 10.3390/biology11020152

**Published:** 2022-01-18

**Authors:** Tobias Lienen, Arne Schnitt, Jens Andre Hammerl, Sven Maurischat, Bernd-Alois Tenhagen

**Affiliations:** German Federal Institute for Risk Assessment (BfR), Department Biological Safety, 10589 Berlin, Germany; Arneschnitt@posteo.de (A.S.); Jens-Andre.Hammerl@bfr.bund.de (J.A.H.); sven.maurischat@bfr.bund.de (S.M.)

**Keywords:** *Mammaliicoccus*, whole-genome sequencing, antimicrobial resistance, dairy farm

## Abstract

**Simple Summary:**

Worldwide, antimicrobial resistance (AMR) is of major concern for human and animal health since infections with multidrug-resistant bacteria are often more challenging and costly. In the family *Staphyloccocaceae*, the species *Staphylococcus* *aureus* in particular was reported to cause severe infections. Although most of the other *Staphylococcaceae* members were not shown to cause severe illnesses, the transmission of AMR genes to harmful species might take place. Therefore, the monitoring of AMR potential in different environments is of high relevance. Mammaliicocci on dairy farms might represent such an AMR gene reservoir. Thus, in this study, the AMR potential of mammaliicocci isolates from German dairy farms was investigated. Whole-genome sequencing (WGS) of the isolates was conducted to evaluate the phylogenetic relationship of the isolates and analyze AMR genes. In addition, antimicrobial susceptibility testing was performed to compare the AMR genotype with the phenotype. It turned out that mammaliicocci may harbor large numbers of different AMR genes and exhibit phenotypic resistance to various antibiotics. Since some AMR genes are likely located on mobile genetic elements, such as plasmids, AMR gene transmission between members of the *Staphylococcaceae* family might occur.

**Abstract:**

Mammaliicocci might play a major role in antimicrobial resistance (AMR) gene transmission between organisms of the family *Staphylococcaceae*, such as the potentially pathogenic species *Staphylococcus aureus*. The interest of this study was to analyze AMR profiles of mammaliicocci from German dairy farms to evaluate the AMR transmission potential. In total, 65 mammaliicocci isolates from 17 dairy farms with a history of MRSA detection were analyzed for AMR genotypes and phenotypes using whole genome sequencing and antimicrobial susceptibility testing against 19 antibiotics. The various genotypic and phenotypic AMR profiles of mammaliicocci from German dairy farms indicated the simultaneous occurrence of several different strains on the farms. The isolates exhibited a non-wildtype phenotype to penicillin (58/64), cefoxitin (25/64), chloramphenicol (26/64), ciprofloxacin (25/64), clindamycin (49/64), erythromycin (17/64), fusidic acid (61/64), gentamicin (8/64), kanamycin (9/64), linezolid (1/64), mupirocin (4/64), rifampicin (1/64), sulfamethoxazol (1/64), streptomycin (20/64), quinupristin/dalfopristin (26/64), tetracycline (37/64), tiamulin (59/64), and trimethoprim (30/64). Corresponding AMR genes against several antimicrobial classes were detected. Linezolid resistance was associated with the *cfr* gene in the respective isolate. However, discrepancies between genotypic prediction and phenotypic resistance profiles, such as for fusidic acid and tiamulin, were also observed. In conclusion, mammaliicocci from dairy farms may carry a broad variety of antimicrobial resistance genes and exhibit non-wildtype phenotypes to several antimicrobial classes; therefore, they may represent an important source for horizontal gene transfer of AMR genes to pathogenic *Staphylococcaceae.*

## 1. Introduction

Bovine mastitis is frequently related to the presence of *Staphylococcus* (*S*.) *aureus*, which is the most prominent species in the family *Staphylococcaceae*. Along with its virulent character, the occurrence of methicillin-resistant *S*. *aureus* (MRSA) complicates the treatment of udder infections. MRSA carries a staphylococcal cassette chromosome (SCC) *mec* element, which harbors the *mecA* or *mecC* gene, transmitting resistance to virtually all beta-lactam antibiotics. Moreover, MRSA may exhibit resistance to various other antibiotics. MRSA and other *Staphylococcaceae* may also be transmitted from animals to humans and vice versa, illustrating their relevance with respect to a One Health approach.

Since antimicrobial resistance (AMR) genes are mostly located on mobile genetic elements such as plasmids or prophages, resistance may be transmitted between different species of the family *Staphylococcaceae*. In particular, organisms of the so-called *S*. *sciuri* group were shown to inhabit various environments [1]. According to phylogenomic analyses, members of this group were recently reassigned to the novel genus *Mammaliicoccus* [2]. The genus consists of the five species *Mammaliicoccus* (*M*.) *sciuri*, *M*. *fleurettii*, *M*. *lentus*, *M*. *vitulinus*, and *M*. *stepanovicii*. These organisms were previously reported to harbor AMR genes against several antibiotics [1] and show unusual SCC*mec* elements, such as a SCC*mec*–*mecC* hybrid element [3,4]. Mammaliicocci were occasionally found on dairy farms and were rarely related to bovine mastitis cases [1,5,6]. However, *mecA* genes harboring *M. sciuri* were recently found to be the most frequently detected species in quarter milk samples on German dairy farms with a history of MRSA detection [6]. In addition, in the same study, beta-lactam antibiotic-resistant *M*. *sciuri* and *M*. *lentus* were the most frequently found species in the nasal swabs of calves [6]. Moreover, mammaliicocci were isolated from human clinical samples, and animal–human contact was considered one feasible transmission route [1]. Next to their potential but rarely expressed pathogenic character, the AMR gene transmission potential of mammaliicocci in particular is of high importance. Recently, a study reported that *M*. *sciuri* served as a *mecA* gene reservoir to *S*. *aureus* isolated from seabirds [7]. In general, beta-lactam resistance most likely originated from non-*aureus Staphylococcaceae*, and native SCC*mec* elements were transmitted to *S*. *aureus*, which further developed into MRSA [8]. Therefore, investigating *Staphylococcaceae* members, such as species of the genus *Mammaliicoccus*, from different environments gives further insights into this topic and reveals alternative AMR mechanisms that might be of public health relevance.

The aim of this study was to characterize the AMR genotype and AMR phenotype of *Mammaliicoccus* isolates from German dairy farms in order to evaluate the resistance potential and risk of AMR gene transmission to more harmful species, such as *S*. *aureus*.

## 2. Materials and Methods

### 2.1. Sample Collection

For this study, 64 *Mammaliicoccus* strains from 17 dairy farms (coded as A-R) located in nine German federal states were chosen for phylogenetic analyses and investigation of their AMR potential. Farms were pre-selected on the basis of a history of MRSA detection in the dairy cow herds [9]. The strains were isolated from milk and swab samples by a two-step selective enrichment with cefoxitin-containing media (3.5 and 4 mg/L). Identification of presumptive mammaliicoccal isolates was carried out by MALDI-TOF analyses (Bruker, Germany) as previously described [6].

### 2.2. Whole-Genome Sequencing and Bioinformatics Analysis

Mammaliicocci isolates were inoculated in 5 mL brain–heart-infusion broth and incubated at 37 °C for 24 h. The DNA of 1 mL culture was extracted using the Qiagen DNeasy Blood and Tissue Kit (Qiagen, Germany) according to the manufacturer’s protocol modified by adding 10 µL lysostaphin to the lysis buffer. The DNA library was prepared using an Illumina DNA Prep kit (Illumina Inc., San Diego, CA, USA), and the 150 bp paired-end sequencing run was performed on an Illumina NextSeq 500 instrument. Raw Illumina reads were trimmed and assembled de novo with the in-house developed AQUAMIS pipeline [10]. Genomic species identification of isolates was performed using the Type Strain Genome Server (TYGS) (https://tygs.dsmz.de/, accessed on 8 August 2021) [11]. Phylogenetic analysis of *M*. *sciuri* and *M*. *lentus* isolates was conducted using CSI Phylogeny 1.4 from the Centre for Genomic Epidemiology (https://cge.cbs.dtu.dk/services/CSIPhylogeny/, accessed on 8 August 2021) [12]. Complete circular genomes of *M*. *sciuri* NCTC12103 strain (NZ_LS483305.1) and *M*. *lentus* NCTC12102 strain (NZ_UHDR01000002.1) were used as reference genomes. Visualization of phylogenetic trees was performed in MEGA X version 10.1.7. Bacterial characterization was conducted with the in-house developed Bakcharak pipeline (https://gitlab.com/bfr_bioinformatics/bakcharak, accessed on 4 March 2021) using the NCBI AMRfinder database [13] for screening of AMR genes. Individual search for the *dfrE* gene (NZ_KZ846041.1) was performed using NCBI BLASTN. Detection of SCC*mec* elements was conducted using SCCmecFinder 1.2 (https://cge.cbs.dtu.dk/services/SCCmecFinder/, accessed on 8 August 2021).

### 2.3. Antimicrobial Susceptibility Testing

Antimicrobial susceptibility testing was performed by broth microdilution according to the CLSI guidelines (ISO 20776-1:2006 or CLSI M31-A3). It was carried out using a standardized antibiotic panel (EUST scheme) that is recommended by the European Food Safety Authority (EFSA) for resistance monitoring in MRSA from livestock and food [14]. For interpretation of the minimum inhibitory concentration (MIC) of the individual isolates, the EUCAST ECOFFs for *S. aureus* were used as follows: PEN, ≤0.125 mg/L; FOX, ≤4 mg/L; CHL, ≤16 mg/L; CIP, ≤1 mg/L; CLI, ≤0.25 mg/L; ERY, ≤1 mg/L; FUS, ≤0.5 mg/L; GEN, ≤2 mg/L; KAN, ≤8 mg/L; LZD, ≤4 mg/L; MUP, ≤1 mg/L; RIF, ≤0.016 mg/L; SMX, ≤128 mg/L; STR, ≤16 mg/L; SYN, ≤1 mg/L; TET, ≤1 mg/L; TIA, ≤2 mg/L; TMP, ≤2 mg/L; VAN, ≤2 mg/L. For quality control of resistance testing, the *S. aureus* isolates ATCC 29213 and ATCC 25923 were used.

## 3. Results

### 3.1. Phylogeny

Presumptive mammaliicoccal isolates were verified by taxonomic assignment in TYGS. The analyzed isolates were further classified as *M*. *sciuri* (26/64), *M*. *lentus* (22/64), *M*. *fleurettii* (15/64), and *M*. *vitulinus* (1/64). However, according to a variable G+C content, the taxonomic assignment of *M*. *fleurettii* isolates seemed to be unreliable; thus, these isolates were named *M*. sp. in the following.

**Figure 1:**
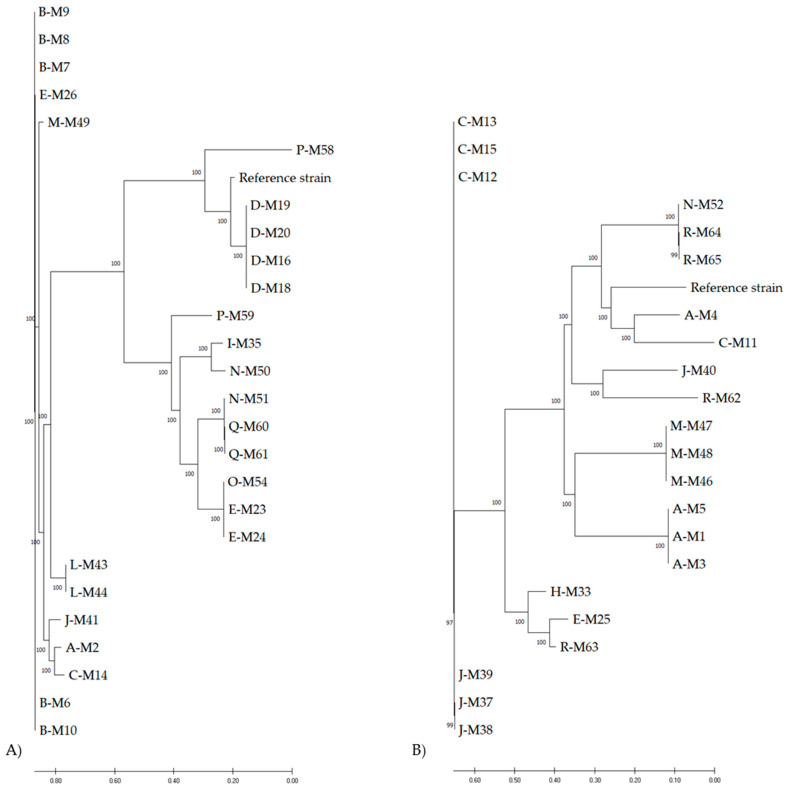
Phylogenetic analyses of *M*. *sciuri* (**A**) and *M*. *lentus* (**B**) isolates from German dairy farms visualized as circular tree using CSI Phylogeny and MEGA X. Letters A–R represent different dairy farms.

Separate phylogenetic trees of *M*. *sciuri* and *M*. *lentus* isolates, which represent the major fractions of the analyzed isolates, show both genomic differences between the dairy farms and closely related strains within distinct farms. *M*. *sciuri* isolates from dairy farm D (M19, M20, M16, and M18) clustered closely in the phylogenetic tree (Figure 1A). On the contrary, *M*. *sciuri* isolates from dairy farm B (M8/M9, M6/M10) were located on two different branches of the phylogenetic tree, indicating genomic distinction. Moreover, isolates from the farms I/N (M35, M50), E/O (M23/24, M54), N/Q (M51, M60/61), and A/C (M2, M14) clustered on the same branch.

With regard to *M*. *lentus* isolates, several genotypically similar strains occurred on farms A (M1, M3, M5), C (M12, M13, M15), J (M37, M38, M39), M (M46, M47, M48), and R (M64, M65) (Figure 1B). Other *M*. *lentus* strains from dairy farm R (M62, M63) were located on different branches in the phylogenetic tree. In addition, isolates from different dairy farms (A/C, J/R, E/R, N/R) clustered on the same branches, respectively.

### 3.2. AMR Genes

A broad range of AMR genes was detected in the genome sequences of *M*. *sciuri* and *M*. *lentus* isolates. Fewer genes were found in *M*. spp and *M*. *vitulinus* genomes (Table 1, Table 2 and Table 3). All isolates (64/64) carried the *mecA* gene. In addition, the *mecC* gene was detected in one *M*. *sciuri* and one *M*. *lentus* isolate. Resistance to several antibiotic classes was transmitted by different genes: aminoglycoside (*aac(6′)Ie*, *aadD1*, *aph(2”)Ia*, *spd*, *str*), glycopeptide (*bleO*), macrolide (*msr(A)*, *mph(C)*, *ermB*, *erm(43)*, *erm(48)*), trimethoprim (*dfrG*, *dfrK*), tetracycline (*tetK*, *tetL*, *tetM*), pleuromutilin–lincosamide–streptogramin (*lnu(A)*, *sal(A)*), oxazolidinone (*cfr*), and phenicol (*catA*, *fexA*).

In 35/64 isolates, the SCC*mec* element was determined as type III with sequence identities of 67–90% to known *S*. *aureus* SCC*mec* element type III. In addition, one *M*. *sciuri* and one *M*. *lentus* isolate harbored a SCC*mec*–*mecC* hybrid element. In the remaining 27 isolates, no SCC*mec* element was detected.

### 3.3. Phenotypic AMR

The phenotypic resistance observed was not always in line with the predicted AMR genes (Table 1, Table 2 and Table 3). The isolates exhibited a non-wildtype phenotype to penicillin (58/64), cefoxitin (25/64), chloramphenicol (26/64), ciprofloxacin (25/64), clindamycin (49/64), erythromycin (17/64), fusidic acid (61/64), gentamicin (8/64), kanamycin (9/64), linezolid (1/64), mupirocin (4/64), rifampicin (1/64), sulfamethoxazole (1/64), streptomycin (20/64), quinupristin/dalfopristin (26/64), tetracycline (37/64), tiamulin (59/64), and trimethoprim (30/64). *M*. *sciuri* was resistant to five to twelve antimicrobial substances, *M*. *lentus* to between eight and eleven. By contrast, *M*. sp. and *M*. *vitulinus* isolates were never resistant to more than four antimicrobials.

## 4. Discussion

The impact of members of the family *Staphylococcaceae* other than *S*. *aureus* on human and animal health, such as bovine mastitis, is rarely investigated. Apart from the species *S*. *haemolyticus* and *S*. *epidermidis*, which were reported to exhibit various virulence factors [15,16], other *Staphylococcaceae* were only occasionally associated with diseases such as bovine mastitis [17]. However, AMR acquisition and transmission is a highly relevant issue in *Staphylococcaceae*. Therefore, the monitoring of AMR in this group is important with respect to treatment options for harmful species, such as *S*. *aureus*.

In this study, the phylogenetic relationship and AMR potential of mammaliicocci from German dairy farms were investigated. Since the visited dairy farms had a history of MRSA detection, it was assumed that mammaliicoccal species might also express distinct AMR profiles.

The *S. sciuri* group was recently reclassified to the new genus *Mammaliicoccus* [2]. The genus *Mammaliicoccus* consists of five species. In this study, *M*. *sciuri*, *M*. *lentus*, and *M*. *vitulinus* were detected in samples from dairy farms. Moreover, several isolates were typed as *M*. *fleurettii* with, however, unreliable species identification results according to a variable G+C content. The phylogenetic analyses of *M*. *sciuri* and *M*. *lentus* isolates showed broad genomic variability in these two species. On the one hand, similar strains were spread in different niches in one dairy farm, but otherwise, different strains were also present in the same farm. Moreover, the close phylogenetic relationship of some strains from different dairy farms indicates transmission of mammaliicocci between dairy farms. A spread of MRSA within and between German dairy farms was assumed in previous studies [18,19]; thus, this might also be the case for mammaliicocci. Introduction of *Staphylococcaceae* strains into dairy farms and spread within the farms may have different causes. Animal trading may contribute to bacterial introduction to the dairy herd and its environment [20]. Insufficient hygiene measures and animal–human contact may contribute to the spread.

The isolates of this study expressed phenotypic resistance to various antimicrobial substances. Impressively, 18/64 isolates showed non-wildtype phenotypes to ≥10 antibiotics. Mammaliicocci harbored AMR genes to several classes of antibiotics [1]. *M*. *sciuri* was also reported as a reservoir of the *mecA* gene for *S*. *aureus* [7]. Moreover, special hybrid SCC*mec* elements harboring *mecA* and *mecC* genes were reported in *M. sciuri* [3,4]. This hybrid element was also detected in one *M*. *sciuri* and one *M*. *lentus* isolate in this study. In general, the detection of SCC*mec* elements using SCCmecFinder was difficult for the analyzed isolates. For most isolates, the prediction showed a SCC*mec* element type III; however, the sequence identity to known SCC*mec* elements type III from *S*. *aureus* was only 67–90%. Low homologies of *M*. *sciuri* SCC*mec* elements in comparison to SCC*mec* elements from *S*. *aureus* were also reported in a review on the evolution of beta-lactam resistance in staphylococci [8]. According to this review, SCC*mec* elements originated from *M*. *sciuri* and were transmitted to *S*. *aureus*. Because of genomic adaptation processes in *S*. *aureus* with regard to stress responses, such as to antibiotics, SCC*mec* elements in *S*. *aureus* evolved, leading to differences in their genomic structure compared to the native SCCmec element in *M*. *sciuri*.

In addition, for one *M*. *vitulinus* and eight *M*. sp. isolates, the SCCmecFinder did not identify any SCC*mec* elements, indicating acquisition of the *mecA* gene independent from any known SCC*mec* element. The *mecA* gene is believed to originate from species of the previous *S*. *sciuri* group [8], and the original *mecA* gene was not located in a SCC*mec* element [8]. Therefore, the corresponding *M*. *vitulinus* and *M*. sp. isolates might still represent this original genomic status. Interestingly, all isolates that lacked an SCC*mec* element exhibited only low cefoxitin MICs of ≤4 mg/L. This indicates susceptibility to beta-lactam antibiotics despite the *mecA* gene. Most likely, the missing accompanying genes in the non-SCC*mec*-*mecA* isolates that are usually present in the SCC*mec* element lead to reduced beta-lactam resistance. Moreover, in general, the *M*. sp. and *M*. *vitulinus* isolates showed only low numbers of AMR genes and, accordingly, exhibited resistance to only a few antimicrobial substances. This might also indicate a native status of these isolates and might illustrate a lower competence of gene acquisition in these species.

Only a few isolates expressed resistance to the aminoglycosides kanamycin (9/64) and gentamicin (8/64), whereas streptomycin resistance was detected in nearly a third of the isolates (20/64). Resistance to kanamycin, gentamicin, and streptomycin was encoded by the *aac(6′)Ie*, *aadD1*, *aph(2”)Ia*, and *str* genes. Resistance to kanamycin and gentamicin mostly co-occurred in the isolates.

Resistance to linezolid is of high public relevance since it belongs to the last-resort antibiotics. Linezolid resistance was detected in one *M*. *sciuri* isolate in this study. Accordingly, the respective isolate harbored a *cfr* gene. Several studies describe the detection of *cfr* genes in members of the family *Staphylococcaceae* [21,22]. The *cfr* gene is usually located on plasmids, which can be transmitted between bacterial species. Thus, it must be considered that the occurrence of the *cfr* gene harboring mammaliicocci on dairy farms with a concurrent MRSA appearance, as presented in this study, may increase the risk of a linezolid resistance acquisition of MRSA.

Macrolide resistance was likely determined by different *erm* genes and the *msr(A)* gene. In particular, *erm* genes play a major role in erythromycin resistance [23]. The *erm* genes were detected in 15/17 of erythromycin-resistant isolates in this study. The two other isolates harbored the *msr(A)* gene in their genomes. The *erm* genes may also confer inducible or constitutive resistance to lincosamides, such as clindamycin [24]. Clindamycin resistance was widely spread in the mammaliicocci isolates of this study (49/64). However, only 15 clindamycin-resistant isolates harbored *erm* genes. In addition, 13 isolates carried the *lnu(A)* gene, which may also provide resistance to lincosamides [25].

Resistance to trimethoprim was mostly associated with *dfrG* or *dfrK* genes (23/30 isolates). Prediction of AMR genes with regard to trimethoprim in the other seven isolates failed. It is likely that these isolates carry variable AMR genes, which are not yet covered by the NCBI AMRfinder database. Recently, a *dfrE* gene, which originates from *Enterococcus faecalis*, was discovered in a multidrug-resistant *M*. *sciuri* strain [26]. However, individual *dfrE* gene search using NCBI BLASTN did not score a hit for this gene in the isolates of this study.

By contrast, the prediction of tetracycline resistance was in good agreement with the respective phenotype. The resistant isolates harbored *tetM*, *tetL*, or *tetK* genes. Tetracycline is often used in livestock farming, increasing the pressure for bacteria to acquire resistance [27]. Although the tetracycline resistance rate of 58% in the mammaliicoccal isolates from this study is rather high, a previous study on MRSA from the same German dairy farms showed even higher tetracycline resistance rates [18].

Interestingly, the tiamulin resistance rate in the mammaliicocci in this study was also very high (92%), exceeding the rate of MRSA from the same German dairy farms [18]. Tiamulin resistance may be transmitted by different genes, such as *sal*, *vga*, and *lsa* genes [28]. However, a respective AMR gene was not predicted for 33/59 of the tiamulin-resistant isolates in this study. Only the *sal(A)* gene was found in a part of the tiamulin-resistant isolates. Thus, it is very likely that tiamulin resistance is additionally affected by a completely different mechanism or encoded by so-far unknown resistance genes, which are not covered by the AMR gene database.

Chloramphenicol resistance was found in 26/64 isolates. Correspondingly, a *fexA* gene was detected in all respective isolates. Moreover, one *M*. *lentus* isolate harbored the *catA* gene, which is also associated with phenicol resistance. However, only a lowered susceptibility of this isolate to chloramphenicol with a MIC of 16 mg/L was detected. The same reduced susceptibility was also found in three other isolates carrying the *fexA* gene.

Although almost all isolates expressed resistance to fusidic acid, a respective AMR gene was not predicted. Fusidic acid resistance may be transmitted by *fus* genes. For other members of the family *Staphylococcaceae*, variations in the *fus* gene had already been detected [29]. However, the variant *fus* gene was not found in the mammaliicoccal isolates of this study. In accordance with the tiamulin resistance, for fusidic acid, it is also reasonable to assume that alternative AMR genes or mechanisms transmitted the phenotypic resistance.

## 5. Conclusions

This study illustrates that mammaliicocci from dairy farms may carry a broad variety of AMR genes and exhibit non-wildtype phenotypes to several antimicrobial classes. It cannot be ruled out that resistance genes are transmitted from mammaliicocci to more pathogenic species of the family *Staphylococcaceae*, such as *S*. *aureus*, which might increase the risk of difficult-to-treat infections in humans and animals. With regard to the One Health approach, resistance to last-resort antibiotics, such as linezolid, is highly concerning. Mismatches of AMR gene prediction and the respective phenotype were occasionally found in the analyzed isolates. As already reported in other studies for different *Staphylococcaceae*, the AMR gene variability in this family is high, and recent databases do not fully cover the variations so far. In order to improve the monitoring of rarely investigated *Staphylococcaceae* genera with regard to the AMR potential, well-curated and complemented AMR gene databases are needed.

## Figures and Tables

**Table 1:** Predicted antimicrobial resistance genes compared to phenotype according to minimum inhibitory concentrations of various antimicrobial substances for the *M. sciuri* isolates. Non-wildtype phenotypes were evaluated according to EUCAST ECOFFs. Grey background represents resistance to respective antimicrobial substance.

Isolate	Species	Not Associated AMR ^1^ Genes	AMR Phenotype ^2^ and Associated AMR Genes																		
			CHL	CIP	CLI	GEN	ERY	FOX	FUS	KAN	LZD	MUP	PEN	RIF	STR	SMX	SYN	TET	TIA	VAN	TMP
A-M2	*M. sciuri*		*fexA*		*erm(B); lnu(A)*	*aac(6’)-Ie;aph(2’’)-Ia*	*erm(B)*	*mecA*		*aac(6’)-Ie;aph(2’’)-Ia*			*mecA*					*tet(L);tet(M)*	*sal(A)*		
B-M6	*M. sciuri*				*erm(B)*		*erm(B)*						*mecA*						*sal(A)*		
B-M7	*M. sciuri*	*aadD1*			*lnu(A)*								*mecA*					*tet(L)*	*sal(A)*		*dfrK*
B-M8	*M. sciuri*	*aadD1*	*fexA*		*lnu(A)*								*mecA*					*tet(L)*	*sal(A)*		*dfrK*
B-M9	*M. sciuri*	*aadD1*	*fexA*		*lnu(A)*								*mecA*					*tet(L)*	*sal(A)*		*dfrK*
B-M10	*M. sciuri*				*erm(B)*		*erm(B)*						*mecA*						*sal(A)*		
C-M14	*M. sciuri*	*bleO*				*aac(6’)-Ie;aadD1;aph(2’’)-Ia*		*mecA*		*aac(6’)-Ie;aadD1;aph(2’’)-Ia*			*mecA*					*tet(L)*	*sal(A)*		*dfrK*
D-M16	*M. sciuri*		*fexA*					*mecA*					*mecA*					*tet(M)*	*sal(A)*		
D-M18	*M. sciuri*		*fexA*					*mecA*					*mecA*					*tet(M)*	*sal(A)*		
D-M19	*M. sciuri*		*fexA*					*mecA*					*mecA*					*tet(M)*	*sal(A)*		
D-M20	*M. sciuri*		*fexA*					*mecA*					*mecA*					*tet(M)*	*sal(A)*		
E-M23	*M. sciuri*												*mecA*		*str*			*tet(L);tet(M)*	*sal(A)*		
E-M24	*M. sciuri*												*mecA*					*tet(L);tet(M)*	*sal(A)*		
E-M26	*M. sciuri*												*mecA*					*tet(L);tet(M)*	*sal(A)*		
I-M35	*M. sciuri*												*mecA*					*tet(L);tet(M)*	*sal(A)*		
J-M41	*M. sciuri*	*fexA*			*erm(45); lnu(A)*		*erm(45)*	*mecA*					*mecA*						*sal(A)*		
L-M43	*M. sciuri*				*lnu(A)*	*aac(6’)-Ie;aph(2’’)-Ia*		*mecA*		*aac(6’)-Ie;aph(2’’)-Ia*			*mecA*					*tet(L);tet(M)*	*sal(A)*		
L-M44	*M. sciuri*				*lnu(A)*	*aac(6’)-Ie;aph(2’’)-Ia*		*mecA*		*aac(6’)-Ie;aph(2’’)-Ia*			*mecA*					*tet(L);tet(M)*	*sal(A)*		
M-M49	*M. sciuri*		*fexA*		*lnu(A)*		*erm(B)*						*blaZ;mecA;mecC2*					*tet(M)*	*sal(A)*		
N-M50	*M. sciuri*				*erm(B)*	*aac(6’)-Ie;aadD1;aph(2’’)-Ia*	*erm(B)*	*mecA*		*aac(6’)-Ie;aadD1;aph(2’’)-Ia*			*mecA*					*tet(L)*	*sal(A)*		
N-M51	*M. sciuri*	*aadD1*	*fexA*										*mecA*					*tet(L)*	*sal(A)*		
O-M54	*M. sciuri*												*mecA*		*str*			*tet(K);tet(M)*	*sal(A)*		
P-M58	*M. sciuri*	*spd*			*lnu(A)*			*mecA*					*mecA*					*tet(M)*	*sal(A)*		
P-M59	*M. sciuri*	*aadD1*			*lnu(A)*								*mecA*					*tet(M)*	*sal(A)*		
Q-M60	*M. sciuri*		*fexA*										*mecA*						*sal(A)*		
Q-M61	*M. sciuri*		*fexA*										*mecA*						*sal(A)*		

^1^ AMR = antimicrobial resistance, ^2^ CHL = chloramphenicol; CIP = ciprofloxacin; CLI = clindamycin; ERY = erythromycin; FOX = cefoxitin; FUS = fusidic acid; GEN = gentamycin; KAN = kanamycin; LZD = linezolid; MUP = mupirocin; PEN = penicillin; RIF = rifampicin; SMX = sulfamethoxazole; STR = streptomycin; SYN = quinupristin/dalfopristin; TET = tetracycline; TIA = tiamulin; TMP = trimethoprim.

**Table 2:** Predicted antimicrobial resistance genes compared to phenotype according to minimum inhibitory concentrations of various antimicrobial substances for the *M. lentus* isolates. Non-wildtype phenotypes were evaluated according to EUCAST ECOFFs. Grey background represents resistance to respective antimicrobial substance.

Isolate	Species	Not Associated AMR ^1^ Genes	AMR Phenotype ^2^ and Associated AMR Genes																		
			CHL	CIP	CLI	GEN	ERY	FOX	FUS	KAN	LZD	MUP	PEN	RIF	STR	SMX	SYN	TET	TIA	VAN	TMP
A-M1	*M. lentus*	*mph(C)*			*erm(B)*	*aac(6’)-Ie;aadD1;aph(2’’)-Ia*	*erm(B)*			*aac(6’)-Ie;aadD1;aph(2’’)-Ia*			*mecA*					*tet(L)*			*dfrG;dfrK*
A-M3	*M. lentus*	*mph(C)*			*erm(B)*		*erm(B)*						*mecA*		*str*						*dfrG*
A-M4	*M. lentus*				*erm(B)*		*erm(B)*			*aac(6’)-Ie;aadD1;aph(2’’)-Ia*			*mecA*					*tet(L)*			*dfrK*
A-M5	*M. lentus*	*mph(C)*			*erm(B)*		*erm(B)*						*mecA*		*str*						*dfrG*
C-M11	*M. lentus*	*catA;lnu(A); mph(C)*											*mecA*		*str*						*dfrG*
C-M12	*M. lentus*	*mph(C)*	*fexA*		*lnu(A)*			*mecA*					*mecA*		*str*						*dfrG*
C-M13	*M. lentus*	*mph(C)*	*fexA*		*lnu(A)*			*mecA*					*mecA*		*str*						*dfrG*
C-M15	*M. lentus*	*mph(C)*	*fexA*		*lnu(A)*			*mecA*					*mecA*		*str*						*dfrG*
E-M25	*M. lentus*	*mph(C)*	*fexA*					*mecA*					*mecA*					*tet(K);tet(M)*			*dfrG*
H-M33	*M. lentus*	*aadD1;mph(C)*	*fexA*		*lnu(A)*			*mecA*					*mecA*					*tet(L)*			*dfrK*
J-M37	*M. lentus*	*mph(C)*	*fexA*		*lnu(A)*			*mecA*					*mecA*					*tet(L)*			*dfrG*
J-M38	*M. lentus*	*mph(C)*	*fexA*		*lnu(A)*			*mecA*					*mecA*					*tet(L)*			*dfrG*
J-M39	*M. lentus*	*mph(C)*	*fexA*		*lnu(A)*			*mecA*					*mecA*		*str*			*tet(K)*			*dfrG*
J-M40	*M. lentus*	*mph(C);spd*	*fexA*		*erm(B)*	*aac(6’)-Ie;aadD1;aph(2’’)-Ia*	*erm(B)*			*aac(6’)-Ie;aadD1;aph(2’’)-Ia*			*mecA*		*str*			*tet(L);tet(M)*			*dfrK*
M-M46	*M. lentus*	*mph(C)*	*fexA*		*erm(43)*		*erm(43)*	*mecA*					*mecA*		*str*			*tet(K);tet(M)*			
M-M47	*M. lentus*	*mph(C)*	*fexA*		*erm(43)*		*erm(43)*	*mecA*					*mecA*		*str*			*tet(K);tet(M)*			
M-M48	*M. lentus*	*mph(C)*	*fexA*		*erm(43)*		*erm(43)*	*mecA*					*mecA*		*str*			*tet(K);tet(M)*			
N-M52	*M. lentus*	*mph(C)*	*fexA*								*cfr*		*mecA*								*dfrG*
R-M62	*M. lentus*	*mmph(C)*	*fexA*		*erm(43); lnu(A)*		*erm(43)*						*blaZ; mecA;mecC2*		*str*			*tet(K);tet(M)*			*dfrG*
R-M63	*M. lentus*	*aadD1;bleO;cfr;mph(C)*	*fexA*					*mecA*					*mecA*		*str*			*tet(K)*			*dfrG*
R-M64	*M. lentus*	*mph(C)*						*mecA*					*mecA*		*str*			*tet(K)*			*dfrG*
R-M65	*M. lentus*	*mph(C)*											*mecA*		*str*			*tet(K)*			*dfrG*

^1^ AMR = Antimicrobial resistance, ^2^ CHL = chloramphenicol; CIP = ciprofloxacin; CLI = clindamycin; ERY = erythromycin; FOX = cefoxitin; FUS = fusidic acid; GEN = gentamycin; KAN = kanamycin; LZD = linezolid; MUP = mupirocin; PEN = penicillin; RIF = rifampicin; SMX = sulfamethoxazole; STR = streptomycin; SYN = quinupristin/dalfopristin; TET = tetracycline; TIA = tiamulin; TMP = trimethoprim.

**Table 3:** Predicted antimicrobial resistance genes compared to phenotype according to minimum inhibitory concentrations of various antimicrobial substances for *M.* sp. and *M. vitulinus* isolates. Non-wildtype phenotypes were evaluated according to EUCAST ECOFFs. Grey background represents resistance to respective antimicrobial substance.

Isolate	Species	Not Associated AMR ^1^ Genes	AMR Phenotype ^2^ and Associated AMR Genes																		
			CHL	CIP	CLI	GEN	ERY	FOX	FUS	KAN	LZD	MUP	PEN	RIF	STR	SMX	SYN	TET	TIA	VAN	TMP
D-M17	*M.* sp.												*mecA*								
E-M21	*M.* sp.	*mph(C)*					*msr(A)*														
E-M22	*M.* sp.	*mph(C)*					*msr(A)*						*mecA*								
F-M27	*M. vitulinus*	*fexA;lnu(A)*													*str*			*tet(K); tet(M)*			
G-M28	*M.* sp.												*mecA*								
G-M30	*M.* sp.												*mecA*								
G-M31	*M.* sp.												*mecA*								
H-M32	*M.* sp.				*lnu(A)*								*mecA*								
H-M34	*M.* sp.												*mecA*								
I-M36	*M.* sp.												*mecA*		*str*						
K-M42	*M.* sp.																				
M-M45	*M.* sp.				*lnu(A)*								*mecA*								
O-M53	*M.* sp.							*mecA*													
P-M55	*M.* sp.																				
P-M56	*M.* sp.	*lnu(A)*											*mecA*								
P-M57	*M.* sp.																				

^1^ AMR = antimicrobial resistance, ^2^ CLI = clindamycin; ERY = erythromycin; FOX = cefoxitin; FUS = fusidic acid; PEN = penicillin; STR = streptomycin; TET = tetracycline; TIA = tiamulin; TMP = trimethoprim.

## Data Availability

The assembled sequences of all strains in this study are deposited in NCBI under BioProject PRJNA641762.

## References

[1] Nemeghaire S., Argudín M.A., Feßler A.T., Hauschild T., Schwarz S., Butaye P. (2014). The ecological importance of the *Staphylococcus sciuri* species group as a reservoir for resistance and virulence genes. Vet. Microbiol..

[2] Madhaiyan M., Wirth J.S., Saravanan V.S. (2020). Phylogenomic analyses of the *Staphylococcaceae* family suggest the reclassification of five species within the genus *Staphylococcus* as heterotypic synonyms, the promotion of five subspecies to novel species, the taxonomic reassignment of five *Staphylococcus* species to *Mammaliicoccus* gen. nov., and the formal assignment of *Nosocomiicoccus* to the family *Staphylococcaceae*. Int. J. Syst. Evol. Microbiol..

[3] Paterson G.K. (2020). Genomic epidemiology of methicillin-resistant *Staphylococcus sciuri* carrying a SCC*mec*-*mecC* hybrid element. Infect. Genet. Evol..

[4] Harrison E.M., Paterson G.K., Holden M.T.G., Ba X., Rolo J., Morgan F.J.E., Pichon B., Kearns A., Zadoks R.N., Peacock S.J. (2013). A novel hybrid SCC*mec*-*mecC* region in *Staphylococcus sciuri*. J. Antimicrob. Chemother..

[5] Khazandi M., Al-Farha A.A., Coombs G.W., O’Dea M., Pang S., Trott D.J., Aviles R.R., Hemmatzadeh F., Venter H., Ogunniyi A.D. (2018). Genomic characterization of coagulase-negative staphylococci including methicillin-resistant *Staphylococcus sciuri* causing bovine mastitis. Vet. Microbiol..

[6] Schnitt A., Lienen T., Wichmann-Schauer H., Tenhagen B.A. (2021). The occurrence of methicillin-resistant non-*aureus* staphylococci in samples from cows, young stock, and the environment on German dairy farms. J. Dairy Sci..

[7] Saraiva M.M.S., de Leon C., Silva N., Raso T.F., Serafini P.P., Givisiez P.E.N., Gebreyes W.A., Oliveira C.J.B. (2021). *Staphylococcus sciuri* as a Reservoir of *mecA* to *Staphylococcus aureus* in Non-Migratory Seabirds from a Remote Oceanic Island. Microb. Drug Resist..

[8] Miragaia M. (2018). Factors Contributing to the Evolution of *mecA*-Mediated beta-lactam Resistance in Staphylococci: Update and New Insights From Whole Genome Sequencing (WGS). Front. Microbiol..

[9] Schnitt A., Lienen T., Wichmann-Schauer H., Cuny C., Tenhagen B.A. (2020). The Occurrence and Distribution of Livestock-Associated Methicillin Resistant *Staphylococcus aureus* ST398 on German Dairy Farms. J. Dairy Sci..

[10] Deneke C., Brendebach H., Uelze L., Borowiak M., Malorny B., Tausch S.H. (2021). Species-Specific Quality Control, Assembly and Contamination Detection in Microbial Isolate Sequences with AQUAMIS. Genes.

[11] Meier-Kolthoff J.P., Göker M. (2019). TYGS is an automated high-throughput platform for state-of-the-art genome-based taxonomy. Nat. Commun..

[12] Kaas R.S., Leekitcharoenphon P., Aarestrup F.M., Lund O. (2014). Solving the Problem of Comparing Whole Bacterial Genomes across Different Sequencing Platforms. PLoS ONE.

[13] Feldgarden M., Brover V., Haft D.H., Prasad A.B., Slotta D.J., Tolstoy I., Tyson G.H., Zhao S., Hsu C.H., McDermott P.F. (2019). Validating the AMRFinder Tool and Resistance Gene Database by Using Antimicrobial Resistance Genotype-Phenotype Correlations in a Collection of Isolates. Antimicrob. Agents Chemother..

[14] European Food Safety Authority (2012). Technical specifications on the harmonised monitoring and reporting of antimicrobial resistance in methicillin-resistant *Staphylococcus aureus* in food-producing animals and food. EFSA J..

[15] Argemi X., Hansmann Y., Prola K., Prevost G. (2019). Coagulase-Negative Staphylococci Pathogenomics. Int. J. Mol. Sci..

[16] Heilmann C., Ziebuhr W., Becker K. (2019). Are coagulase-negative staphylococci virulent?. Clin. Microbiol. Infect..

[17] De Buck J., Ha V., Naushad S., Nobrega D.B., Luby C., Middleton J.R., De Vliegher S., Barkema H.W. (2021). Non-*aureus* Staphylococci and Bovine Udder Health: Current Understanding and Knowledge Gaps. Front. Vet. Sci..

[18] Lienen T., Schnitt A., Hammerl J.A., Maurischat S., Tenhagen B.A. (2020). Genomic Distinctions of LA-MRSA ST398 on Dairy Farms From Different German Federal States With a Low Risk of Severe Human Infections. Front. Microbiol..

[19] Lienen T., Schnitt A., Cuny C., Maurischat S., Tenhagen B.-A. (2021). Phylogenetic Tracking of LA-MRSA ST398 Intra-Farm Transmission among Animals, Humans and the Environment on German Dairy Farms. Microorganisms.

[20] Schnitt A., Tenhagen B.A. (2019). Risk Factors for the Occurrence of Methicillin-Resistant *Staphylococcus aureus* in Dairy Herds: An Update. Foodborne Pathog. Dis..

[21] Cui L., Wang Y., Li Y., He T., Schwarz S., Ding Y., Shen J., Lv Y. (2013). *Cfr*-mediated linezolid-resistance among methicillin-resistant coagulase-negative staphylococci from infections of humans. PLoS ONE.

[22] Cuny C., Arnold P., Hermes J., Eckmanns T., Mehraj J., Schoenfelder S., Ziebuhr W., Zhao Q., Wang Y., Fessler A.T. (2017). Occurrence of *cfr*-mediated multiresistance in staphylococci from veal calves and pigs, from humans at the corresponding farms, and from veterinarians and their family members. Vet. Microbiol..

[23] Weisblum B. (1995). Erythromycin Resistance by Ribosome Modification. Antimicrob. Agents Chemother..

[24] Leclercq R. (2002). Mechanisms of Resistance to Macrolides and Lincosamides: Nature of the Resistance Elements and Their Clinical Implications. Clin Infect Dis.

[25] Schwarz S., Feßler A.T., Loncaric I., Wu C., Kadlec K., Wang Y., Shen J. (2018). Antimicrobial Resistance among Staphylococci of Animal Origin. Microbiol. Spectr..

[26] Gómez-Sanz E., Haro-Moreno J.M., Jensen S.O., Roda-García J.J., López-Pérez M. (2021). The Resistome and Mobilome of Multidrug-Resistant *Staphylococcus sciuri* C2865 Unveil a Transferable Trimethoprim Resistance Gene, Designated *dfrE*, Spread Unnoticed. mSystems.

[27] Granados-Chinchilla F., Rodriguez C. (2017). Tetracyclines in Food and Feedingstuffs: From Regulation to Analytical Methods, Bacterial Resistance, and Environmental and Health Implications. J. Anal. Methods Chem..

[28] Wendlandt S., Kadlec K., Fessler A.T., Schwarz S. (2015). Identification of ABC transporter genes conferring combined pleuromutilin-lincosamide-streptogramin A resistance in bovine methicillin-resistant *Staphylococcus aureus* and coagulase-negative staphylococci. Vet. Microbiol..

[29] Chen H.J., Hung W.C., Lin Y.T., Tsai J.C., Chiu H.C., Hsueh P.R., Teng L.J. (2015). A novel fusidic acid resistance determinant, *fusF*, in *Staphylococcus cohnii*. J. Antimicrob. Chemother..

